# Treatment of hepatocellular carcinoma with a GPC3-targeted bispecific T cell engager

**DOI:** 10.18632/oncotarget.17905

**Published:** 2017-05-16

**Authors:** Yanyu Bi, Hua Jiang, Peng Wang, Bo Song, Huamao Wang, Xianming Kong, Zonghai Li

**Affiliations:** ^1^ State Key Laboratory of Oncogenes and Related Genes, Shanghai Cancer Institute, Renji Hospital, Shanghai Jiaotong University School of Medicine, Shanghai, China; ^2^ CARsgen Therapeutics, Shanghai, China; ^3^ Renji Hospital, Shanghai Jiaotong University School of Medicine, Shanghai, China

**Keywords:** hepatocellular carcinoma, glypican-3, bispecific T cell engager, immunotherapy

## Abstract

There are limited strategies for the treatment of hepatocellular carcinoma (HCC). In this study, we prepared a Bispecific T cell engager (BiTE) targeting Glypican 3 (GPC3) and CD3. The GPC3/CD3 BiTE was prepared by fusing the single-chain variable fragment (scFv) of the humanized anti-GPC3 antibody (9F2) with the scFv of the anti-CD3 antibody (OKT3). The *in vitro* and *in vivo* cytotoxic activities of the GPC3/CD3 BiTE were evaluated against various HCC cell lines. The GPC3/CD3 BiTE could efficiently mediate the T cell killing of GPC3-positive HCC *in vitro*, which was dependent on GPC3 expression on the surface of HCC cells. Moreover, our study indicates that, in the presence of the GPC3/CD3 BiTE, T cells could efficiently destroy GPC3-positive human HCC cells *in vitro* and *in vivo*. Additionally, our study further proved that GPC3 is not expressed in normal tissues. Thus, GPC3 may be a cancer-specific antigen. Collectively, these findings suggest that this anti-GPC3 BiTE might be a promising anti-tumor reagent for patients with GPC3-positive HCC.

## INTRODUCTION

Hepatocellular carcinoma is the sixth most common cancer in the world, and the third most frequent cause of cancer-related death [1–2]. The incidence of HCC is quickly increasing in both Asian and Western countries [3]. Currently, surgery is the most effective treatment for HCC. However, tumor recurrence after a curative liver resection is very high and there is only a 10% 5-year survival rate [4]. Moreover, the majority of patients with HCC are diagnosed at a late stage when potentially curative therapies, including chemotherapy, chemoembolization, ablation, and proton beam therapy, are least effective. Sorafenib (Nexavar), the first clinically approved targeted drug therapy for HCC, could only extend overall survival by 2–3 months [4–5]. Additionally, many patients permanently withdraw from treatment because of severe skin toxicity [6]. Thus, there remains an unmet need for tolerable, life-prolonging strategies for patients in the management of HCC.

Bispecific T cell engagers (BiTEs) have shown great promise for the immunological treatment of cancer [7–8]. This structure has been explored as a new means to recruit cytotoxic T cells to kill tumor cells. Target antigens explored for tumor therapy include differentiation antigens such as CD19, CD33, CEA, EpCAM, HER-2/neu, PSMA, and EGF receptor [9–10]. The first BiTE antibody, anti-CD19-CD3 BiTE blinatumomab (Blincyto™), was approved by the FDA in 2014 for the treatment of patients with Philadelphia chromosome-negative relapsed/refractory B cell precursor ALL [11–12], suggesting that BiTE is indeed a promising strategy for the treatment of cancer. Previously, we reported that EpCAM/CD3 BiTE could eliminate HCC cells *in vitro* and *in vivo* [13], suggesting that BiTE might be an alternative method to treat HCC patients. However, EpCAM expression was reported in several normal tissues, including small intestine, colon, lower respiratory tract, trachea, bronchi, bronchioles, alveoli, liver, bile ducts, pancreas, skin, endocrine glands, mammary glands, and the female genital tract [14]. In addition, several anti-EpCAM antibodies have shown significant toxicity in clinical studies [15–16]. Moreover, one patient died of on-target, off-tumor toxicity after treatment with Her-2-redirected chimeric antigen receptor engineered T cells [17]. Thus, we propose that it will be much safer to use another target that has tumor-restricted expression.

Glypican 3 (GPC3), which belongs to the glypican family, is a heparin sulfate proteoglycan and is expressed on the cell surface via a glycerol-phosphatidylinositol (GPI) anchor [18–20]. GPC3 is expressed in a wide range of tissues during development, such as in the placenta and embryonic tumors (Wilms tumor), but its expression is suppressed in most adult tissues, generally through the methylation of DNA within the promoter region [20–21]. Although several studies have indicated that GPC3 is absent in normal tissues, studies by Daniel Baumhoer [22] revealed that most normal tissues stained negatively for GPC3 but that gastric glands (3/7 [43%]), kidney tubules (9/17 [53%]), and testicular germ cells (2/16 [13%]) stained positively for GPC3. However, our study revealed that GPC3 is not expressed in either gastric glands or kidney tissue; we also demonstrated its expression in approximately 70% of HCC and 63% of squamous non-small cell lung cancer [23–24]. More importantly, no severe toxicities were observed in the clinical trials for a GPC3 vaccine and anti-GPC3 monoclonal antibody [20]. Thus, we propose that GPC3 is a rational target for BiTE antibodies.

In this study, a GPC3/CD3 BiTE was prepared, and its inhibitory activities towards HCC were characterized both *in vitro* and *in vivo*.

## RESULTS

### Expression and purification of GPC3/CD3 BiTE

**Figure 1:**
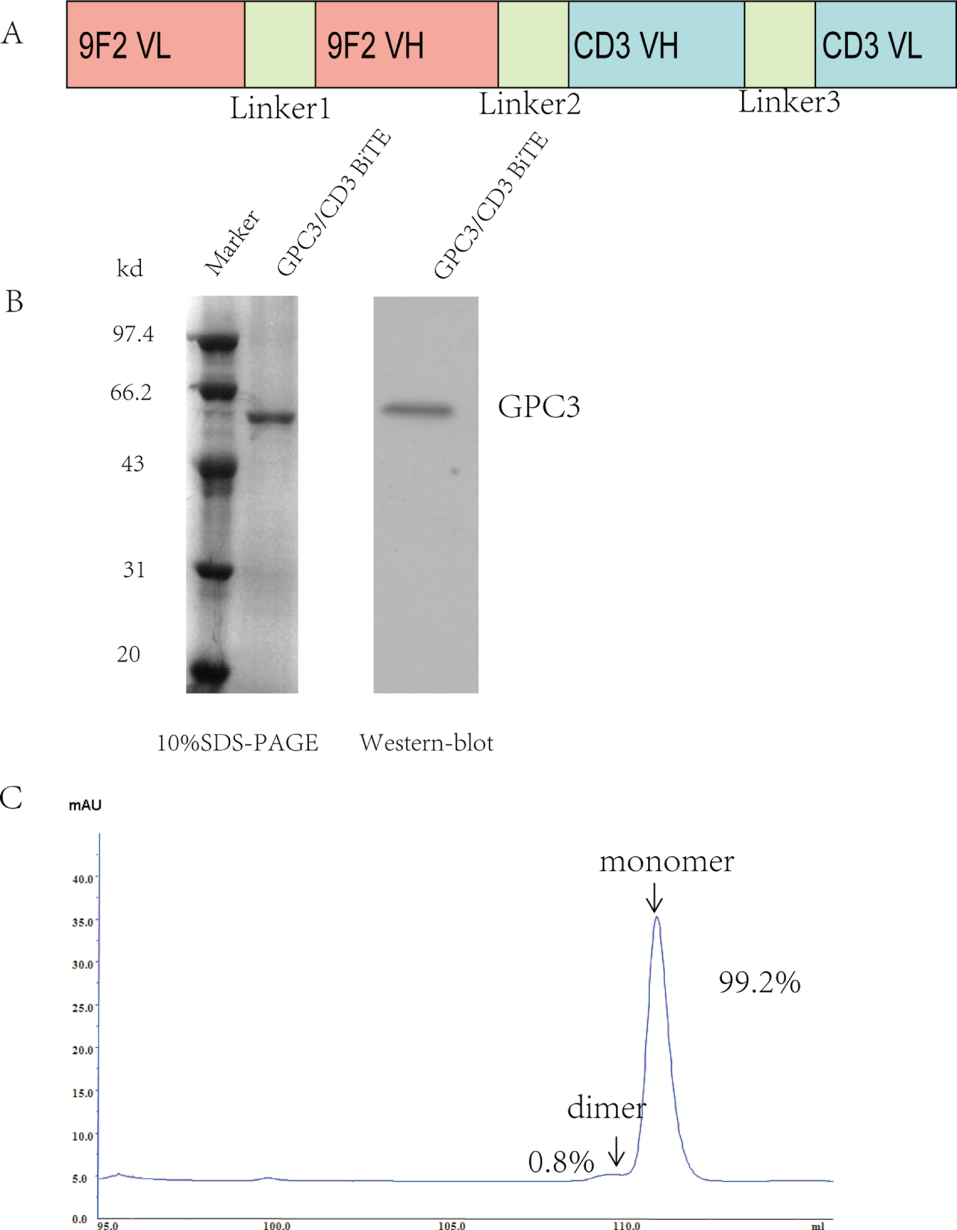
Expression and purification of the GPC3/CD3 BiTE (**A**) Schema of the GPC3/CD3 BiTE design. Linkers L1 and L3 were constructed between the VH and VL domains of the anti-GPC3 scFvs fragment Hu9F2 and the anti-CD3 scFvs and consisted of (Gly_4_Ser)_3_. L2 was constructed as a single Gly_4_Ser linker between these scFvs. (**B**) Coomassie blue-stained 10% SDS-PAGE gel of purified GPC3/CD3 BiTE (lane 2). Lane 1 shows molecular size standards with their apparent molecular weights (Marker) in kiloDaltons (kDa). Western blot at the right of figure B shows that GPC3/CD3 BiTE has a 54-kDa weight. We used an anti-His antibody. (**C**) By SEC-HPLC peak integration, the GPC3/CD3 monomer segregation was estimated to be 99.2% of the total purified protein.

The GPC3/CD3 BiTE was constructed using standard DNA recombination technologies; the scheme is shown in Figure 1A. The GPC3-specific scFv fragment Hu9F2 was fused by a flexible peptide linker to the CD3 scFv fragment, and a His-tag was added. As shown in Figure 1B, the molecular mass of the GPC3/CD3 BiTE was approximately 54 kDa, as analyzed by SDS-PAGE and western blot. The purity of the GPC3/CD3 monomer isolated by molecular sieve approached 99.2%, as assessed by SEC-HPLC (Figure 1C).

### GPC3/CD3 BiTE effectively binds to PBMCs and GPC3^+^ HCC cells

**Figure 2:**
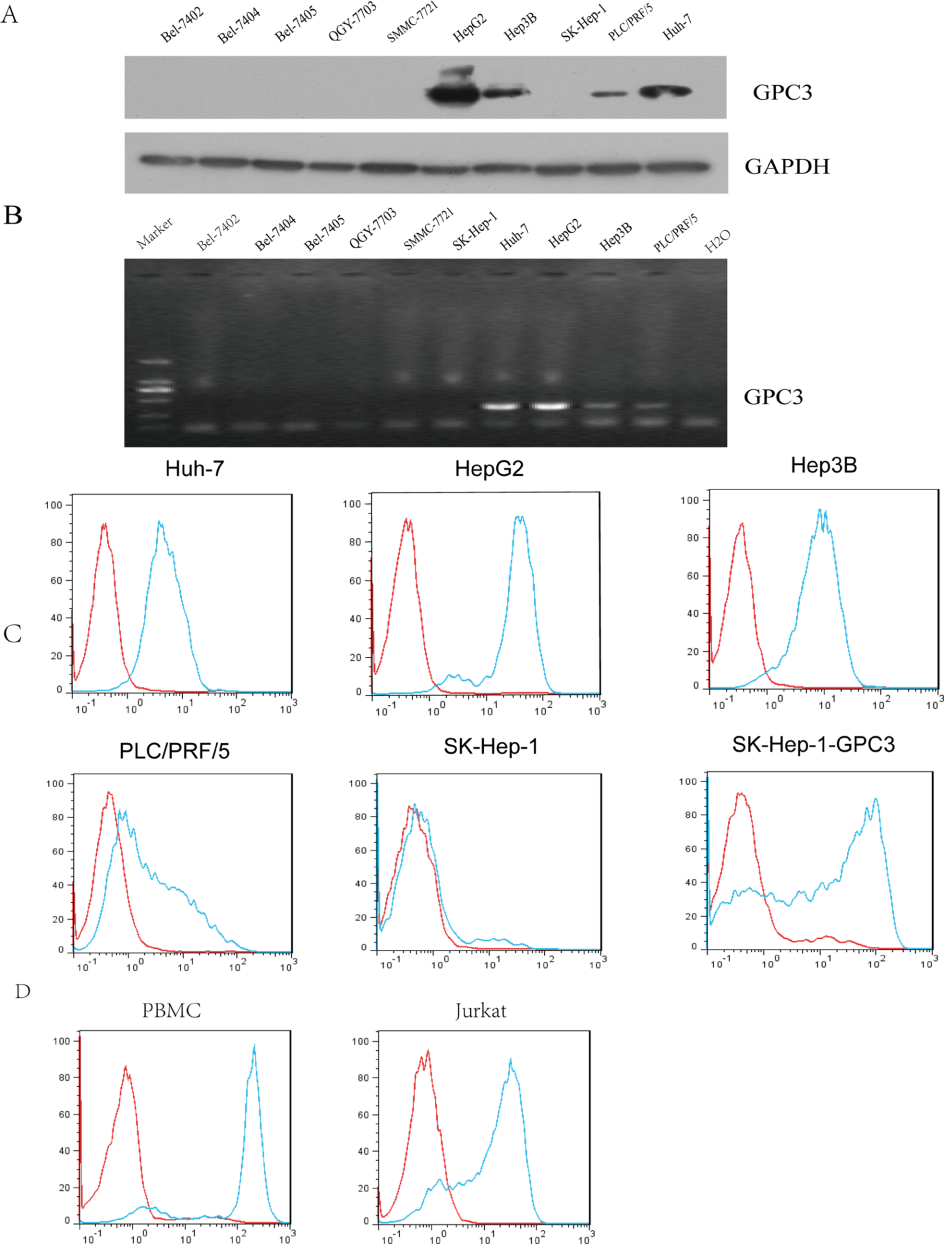
Detection of surface GPC3 expression Surface GPC3 expression in various HCC cell lines was analyzed by western blot analysis. (**A**) and RT-PCR (**B**). We also confirmed the GPC3/CD3 BiTE binding potency with these HCC target cells using flow cytometry; the antibody (green) and isotype antibody control (red) are shown in (**C**) Additionally, the binding with effector cells of GPC3/CD3 BiTE is shown; the effector cells were PBMCs and Jurkat cells, which both express CD3.

Western blot and reverse-transcription–polymerase chain reaction (RT-PCR) assay were used to evaluate the expression of GPC3 in 10 HCC cell lines. The results in Figure 2A, 2B indicated that HepG2, Hep3B, PLC/PRF/5 and Huh-7 cells expressed GPC3 (HepG2>Huh-7>Hep3B>PLC/PRF/5). There was no GPC3 expression in the other six cell lines, including SK-Hep-1. FACS analysis revealed that GPC3/CD3 BiTE strongly bound to SK-Hep-1-GPC3 (GPC3-transfected SK-Hep-1 cell line), HepG2, Huh-7 and Hep3B cells, barely bound to SK-Hep-1 cells and weakly bound to PLC/PRF/5 cells (Figure 2C). We also estimated GPC3 expression in normal human tissues (Supplementary Figure 3). In addition, GPC3/CD3 BiTE could bind efficiently to PBMCs and Jurkat cells expressing CD3. (Figure 2D). This result indicated that the binding specificity of anti-GPC3 BiTE is dependent on the membrane level of GPC3. We also tested the CHO-K1 overexpression system as a positive control of the SK-Hep-1-GPC3 construct by western blot and FACS data, as shown in Supplementary Figure 1B.

### Cytokines released by GPC3/CD3 BiTE-redirected T cells

**Figure 3:**
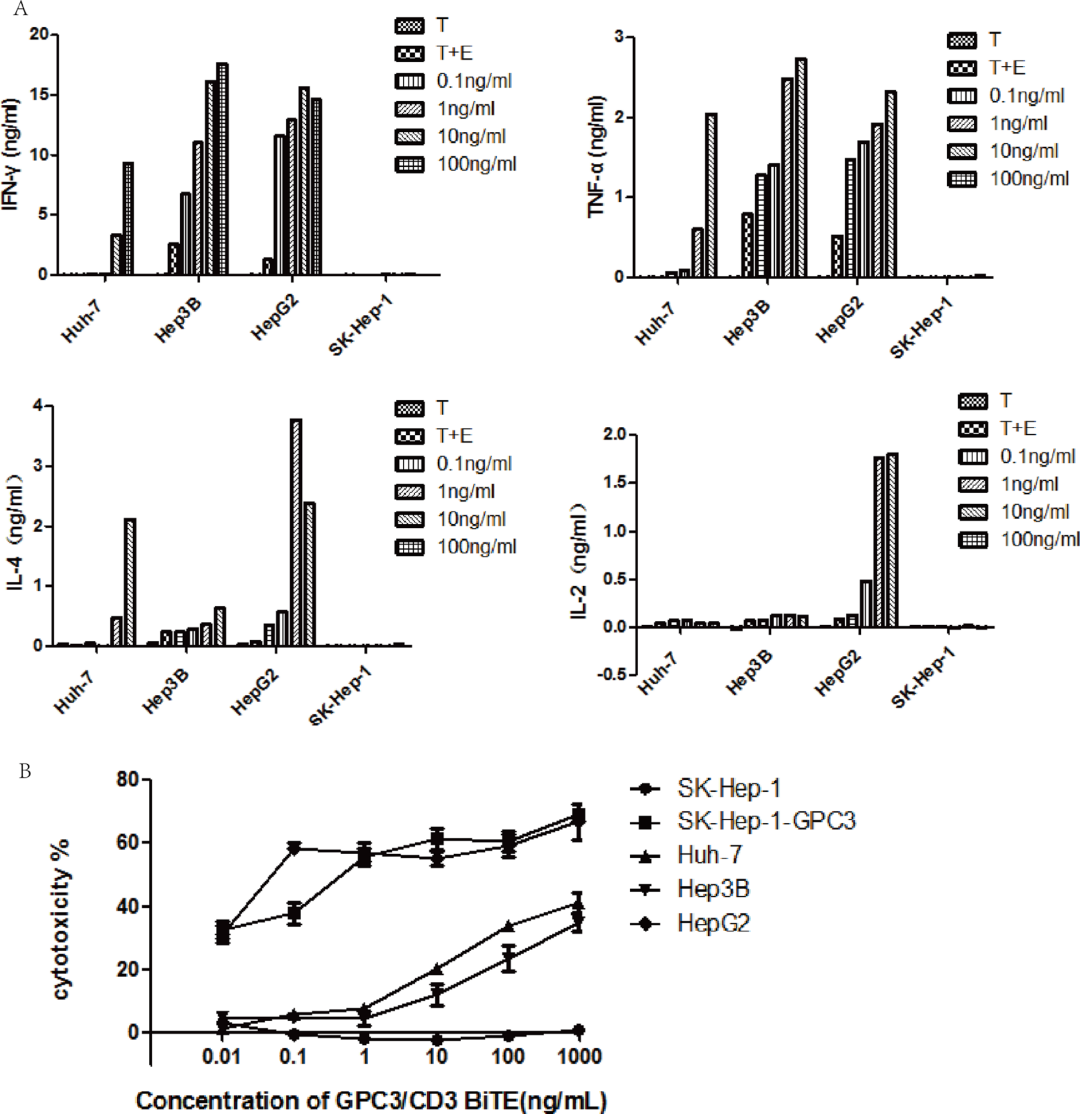
Cytokines released by GPC3/CD3 BiTE-redirected T cells and their cytotoxicity (**A**) Cytokines released after GPC3/CD3 BiTE-redirected T cell lysis in various cocultured HCC cell lines for 40 h; the E: T ratio was 10:1, and the experiments were performed in triplicate. The target cells were Huh-7, Hep3B, HepG2 and SK-Hep-1 cultured with or without the GPC3/CD3 BiTE. (**B**) *In vitro* cytotoxicity of the GPC3/CD3 BiTE redirected T cells. The target cells were Huh-7, Hep3B, HepG2 and SK-Hep-1 and SK-Hep-1 GPC3.

To demonstrate the effect of GPC3/CD3 BiTE on T cellactivation, cytokine release was measured using ELISA, with PBMCs as the effector cells and a range of concentrations of GPC3/CD3 BiTE from 0.1 ng/mL to 100 ng/mL. As shown in Figure 3A, in the presence of GPC3-positive target cells and 10 ng/mL or 100 ng/mL of GPC3 BiTE antibodies, human PBMCs secreted significantly higher concentrations of TNF-α, IFN-γ, and IL-4 into the cell culture supernatant. By contrast, these cytokines were barely detectable in the absence of the BiTE or in the presence of GPC3-negative target cells. Additionally, in the presence of GPC3-positive target cells, 10 ng/mL and 100 ng/mL of the BiTE induced more cytokine (TNF-α, IFN-γ, IL-4) secretion than did the lower concentration of BiTE. Moreover, although much less IL-2 was secreted by the T cells in the presence of Huh-7 and Hep3B, we did observe IL-2 expression in the presence of HepG2 cells, which had the highest level of GPC3 expression. The results indicated that anti-GPC3 BiTE could stimulate T cells to secrete cytokines in the presence of GPC3-positive HCC cells. The concentration of GPC3/CD3 BiTE, as well as the GPC3 expression level in target cells, can affect this stimulation.

### Potent redirected lysis of GPC3^+^ HCC cells by GPC3/CD3 BiTE-activated T cells

The redirected lysis mediated by T cells cocultured with GPC3^+^ HCC cells was investigated in the presence of GPC3/CD3 BiTE. As shown in Figure 3B, the cytotoxicity assay used unstimulated PBMCs from healthy human donors as effector cells at an effector-to-target (E: T) ratio of 10:1 and 18 h of coculture. In the presence of all four GPC3+ HCC cells, the GPC3/CD3 BiTE showed significant cytotoxicity at a very low concentration (1 to 10 ng/mL). Furthermore, in the presence of HepG2 and SK-HEP-1 GPC3 cells, which have higher levels of GPC3 expression than the other target cells, specific lysis could be obviously observed even at a very low concentration (0.01 ng/mL) of the BiTE. By contrast, no specific lysis was observed in SK-HEP-1, the GPC3-negative HCC cell line. Thus, the lysis of cytotoxicity was strictly dependent on the ability of recognition and binding of GPC3/CD3 BiTE on target HCC cells.

### Upregulation of granzyme B in GPC3/CD3 BiTE-redirected T cells

**Figure 4:**
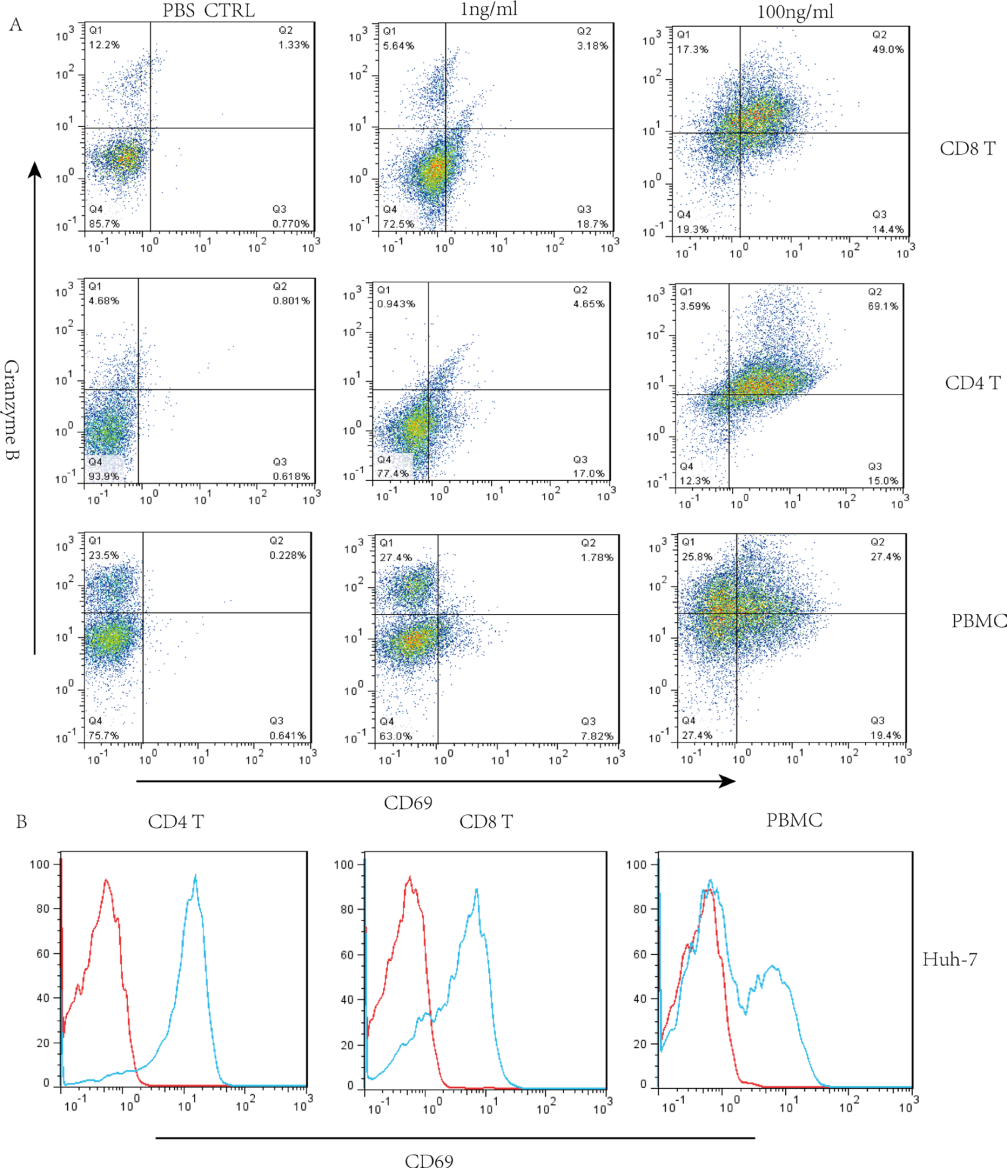
Granzyme B expression when GPC3/CD3 BiTE redirected lysis to HCC in the presence of CD4^+^ and CD8^+^ T cells (**A**) Three-color flow cytometry analysis of granzyme B, PBMCs, CD4^+^ and CD8^+^ T cells separately cocultured with Huh-7 cells in the presence of GPC3/CD3 BiTE. (**B**) We also detected the expression of the activation marker CD69 in permeabilized CD4^+^ and CD8^+^ T lymphocytes in response to incubation with 100 ng/mL of GPC3/CD3 BiTE for 16 h.

It has been reported that the granzyme/perforin pathway plays a significant role in lymphocyte-mediated killing [25], therefore, we investigated the effect of GPC3/CD3 BiTE activity on intracellular expression of granzyme B in CD4^+^ and CD8^+^ T cells. The granzyme B expression in the indicated T cells was enhanced in the presence of both Huh-7 and HepG2 cells at a GPC3/CD3 BiTE concentration of 100 ng/mL (Figure 4A and Supplementary Figure 2A). Additionally, the activation marker CD69 was upregulated in CD4^+^ and CD8^+^ T cells in the presence of the BiTE with both Huh-7 and HepG2 cells (Figure 4B and Supplementary Figure 2B).

### In *vivo* antitumor efficacy for early prevention in three mouse models

**Figure 5:**
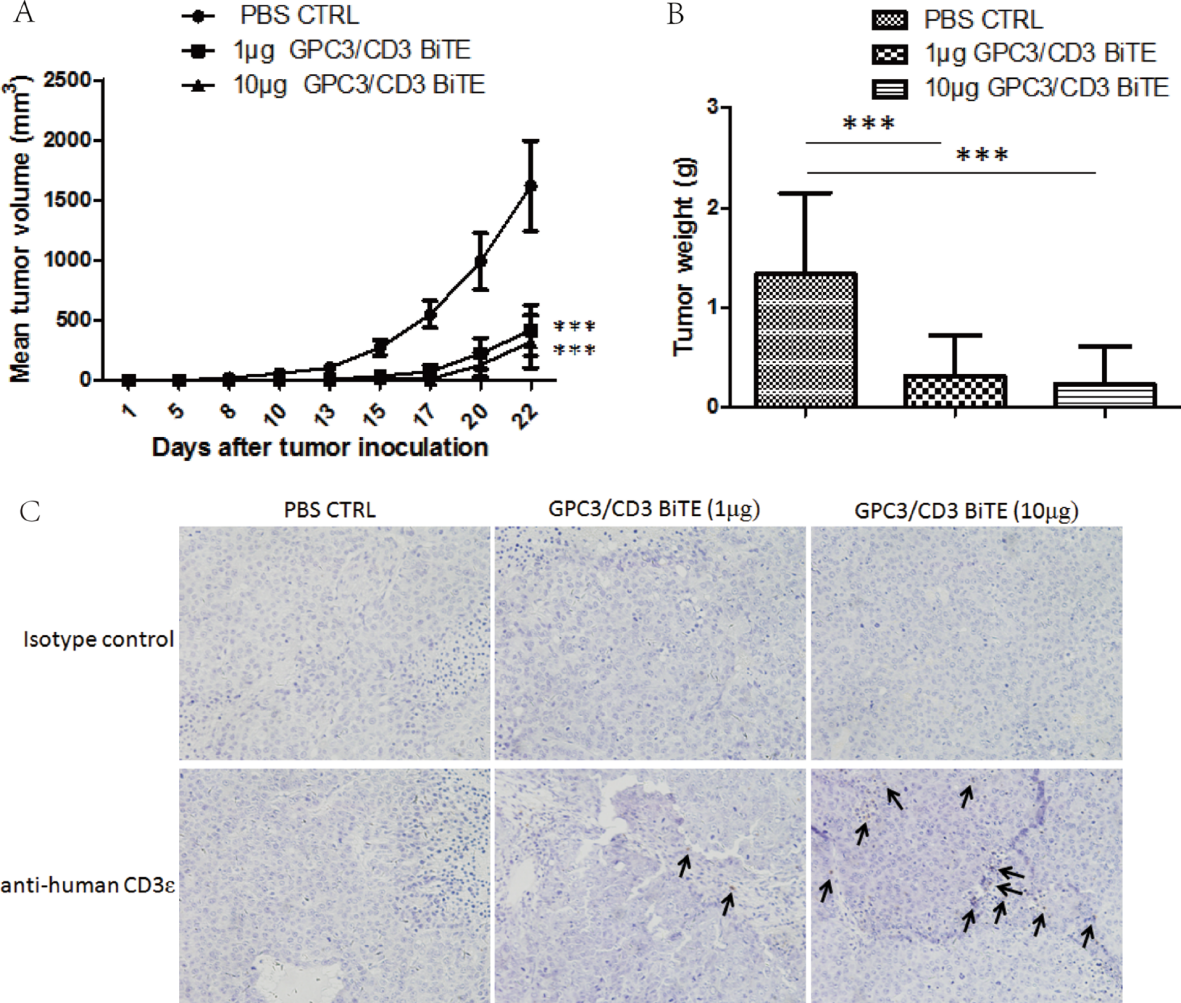
*In vivo* efficacy of GPC3/CD3 BiTE in Huh-7 subcutaneous xenograft models Cohorts of six NOD/SCID mice were used. (**A**) and (**B**) Mice were subcutaneously inoculated with 2 × 10^6^ Huh-7 human HCC cells in the absence or presence of 4 × 10^6^ unstimulated human PBMCs from healthy donors (E:T = 2:1). Animals were either treated through tail vein injection with a PBS vehicle control or with 1 μg or 10 μg GPC3/CD3 BiTE per mouse per day. The mean values of the tumor growth curves are shown for mice treated with the PBS vehicle control in the absence or presence of human PBMC. For Huh-7 treated animals, individual tumor growth curves are shown. (B) Tumor weights were measured when the mice were sacrificed. (***P < 0.001). (**C**) The infiltration of T cells in Huh-7 xenograft tumors. Tumors were collected from mice bearing Huh-7 subcutaneous xenografts treated with PBS, 1 μg GPC3/CD3 BiTE or 10 μg GPC3/CD3 BiTE. Formalin-fixed, paraffin-embedded tumor sections were consecutively cut and stained for human CD3 expression (brown). The images were taken with the microscope (BX41, Olympus, PA) and camera (DP70) under ×200 magnifications.

To explore the *in vivo* antitumor activities of GPC3/CD3 BiTE, Huh-7 cells mixed with unstimulated fresh human PBMCs at an E: T ratio of 2:1 was inoculated into the mice. Treatment began 1 h after the cell mixture was inoculated, and treatment continued for 1–10 days at a dose of 10 μg/intravenous injection of GPC3/CD3 BiTE. At the end of this study (22 days), the results shown in Figure 5A, 5B indicated that 1 μg and 10 μg/injection of GPC3/CD3 BiTE potently suppressed tumor growth.

**Figure 6:**
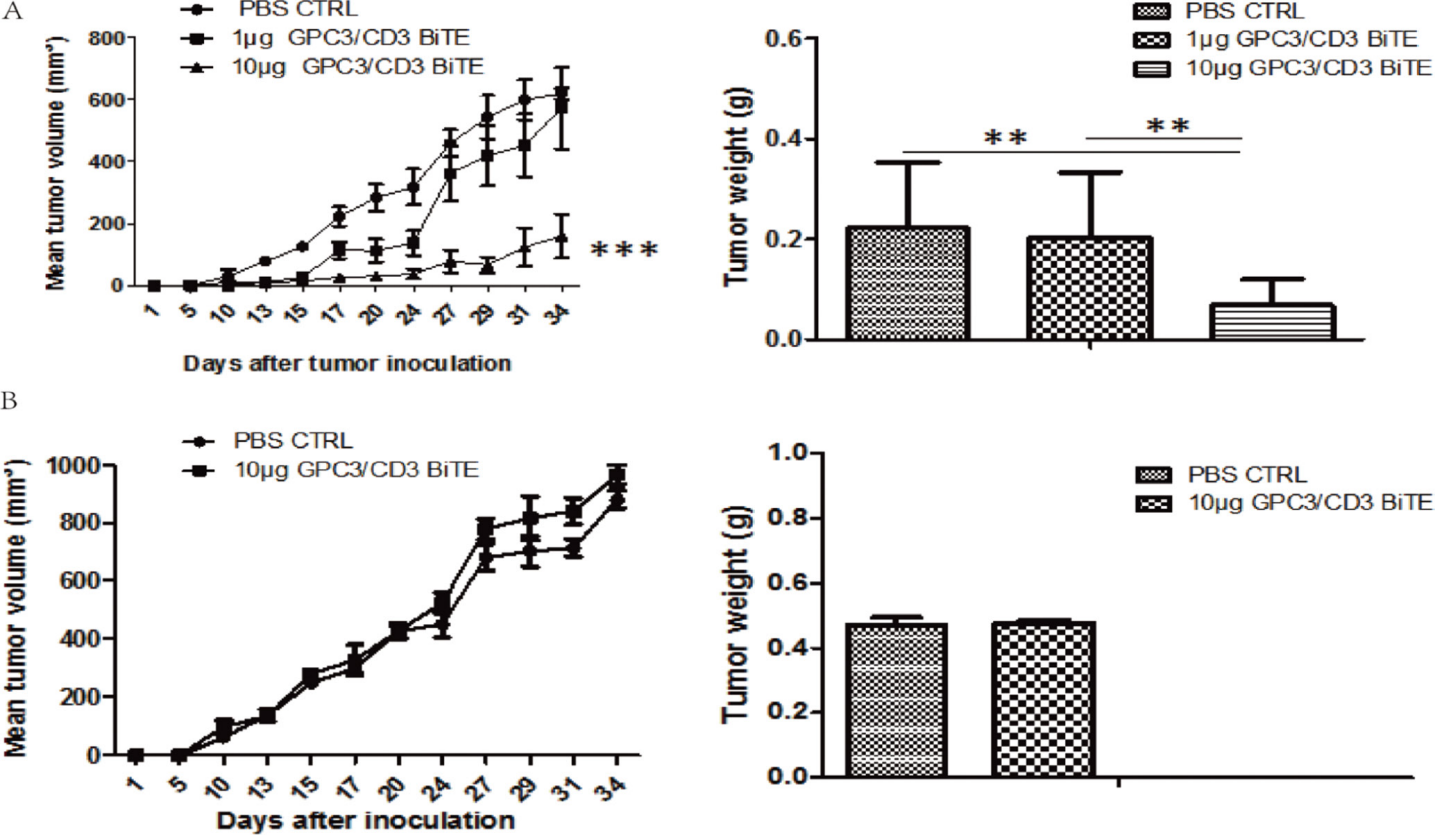
*In vivo* efficacy of GPC3/CD3 BiTE in SK-Hep-1 GPC3 and SK-Hep-1 xenograft models There were six mice in each group. A total of 2 × 10^6^ SK-Hep-1 GPC3 (**A**) or SK-Hep-1 (**B**) cells and 4 × 10^6^ unstimulated human PBMCs were inoculated into the mice. Then, 10 μg or 1 μg of the GPC3/CD3 BiTE or PBS (control) was infused via the tail vein for 5 continuous days. On day 36, the tumors in the 10 μg group were smaller than those in the control group in the SK-Hep-1 GPC3 model (A). In the SK-Hep-1 model (B), there was no difference between the two groups. (***P < 0.001, **P < 0.05)

To further validate that the *in vivo* antitumor activities of the anti-GPC3 BiTE are target dependent, SK-Hep-1 and SK-Hep-1-GPC3 were applied in the *in vivo* antitumor assays. The results indicated that the 10 μg dose of GPC3/CD3 BiTE could potently suppress the outgrowth of SK-Hep-1-GPC3 tumor xenografts *in vivo* (Figure 6A), while both 1 μg and 10 μg doses of GPC3/CD3 BiTE had no effect on the outgrowth of SK-Hep-1 tumor xenografts (Figure 6B). Thus, the anti-GPC3 BiTE has target-dependent antitumor activities *in vivo*.

The infiltration of human T cells was further detected in Huh-7 xenografts tumors treated with GPC3/CD3 BiTE by staining with human CD3 antibodies. The results showed that human CD3^+^ T cells had infiltrated into residual tumors 2 weeks after GPC3/CD3 BiTE administration (Figure 5C). Compared with the infiltrated T cells in tumors treated with 10 μg GPC3/CD3, only fewer T cells could be detected in the tumors treated with 1 μg doses of GPC3/CD3. There was no specific staining in the sections treated with PBS.

## DISCUSSION

Our previous study and the data from the present study indicated that GPC3 is not expressed in any normal tissues examined and is a cancer-specific antigen (Supplementary Figure 3). Additionally, we have also demonstrated that GPC3-redirected T cells (CAR-T) cells could eradicate GPC3-positive HCC xenografts [23], and we have launched a first-in-human study on GPC3-redirected CAR-T cells (NCT02395250). Thus, to elucidate whether an anti-GPC3 BiTE could also eliminate GPC3-positive cancer cells in an antigen-dependent manner, in this study, we performed *in vitro* and *in vivo* experiments. The data here solidly demonstrate that anti-GPC3 BiTE has strong target-dependent antitumor activities against HCC cell lines *in vitro* and *in vivo*.

Although CAR-T cells and BiTEs have some similarities, there are at least two major differences. One difference is that CAR-T cells should be prepared for each specific individual. By contrast, BiTEs can be universally applied. Thus, CAR-T treatment is relatively expensive. The other difference is that lymphodepletion is generally a precondition for CAR-T grafting *in vivo*, while BiTEs do not require lymphodepletion. One major shortcoming of BiTEs is that their half-life is relatively short and, greater administration is needed [26–28]. Because of their small size (55 kDa) and the lack of an Fc domain, BiTEs have a relatively short half-life of a few hours. There are several strategies that may partly overcome this problem. One is using other bispecific antibody formats, such as CrossMab [29]. Another is using other methods, such as PEGylation, to extend the half-life. Continuous intravenous (cIV) infusion by a portable mini-pump may also solve this problem [12].

It is well known that GPC3 is not only expressed in HCC but also expressed in squamous non-small cell lung cancer, esophageal cancer and other cancer types [30–33]. We have demonstrated that GPC3-redirected CAR-T cells could also have potent antitumor activities in other cancer types in addition to HCC [24]. Thus, we propose that the anti-GPC3 BiTE will have similar antitumor activities on these GPC3-positive cancer types. Together, our studies clearly indicate that anti-GPC3 is a promising anti-tumor reagent, especially for patients with HCC.

## MATERIALS AND METHODS

### Cell lines

The human HCC cell lines (HepG2, Hep3B, PLC/PRF/5, and SK-Hep-1), 293T and CHO-K1 were purchased from the American Type Culture Collection. The Huh-7 cell line was obtained from the RIKEN Cell Bank. The 293F cells were obtained from Invitrogen. SK-Hep-1-GPC3 (SK-Hep-1 cells with GPC3 overexpression) and CHO-K1-GPC3 were established by our laboratory. The SMMC-7721, Bel-7404, Bel-7402, Bel-7405 and QGY-7703 HCC cell lines were obtained from the Chinese Academy of Sciences and preserved by our laboratory. HCC cell lines, 293T and CHO-K1 were cultured in Dulbecco’s modified Eagle’s medium (DMEM) supplemented with 10% fetal bovine serum and 1% antibiotics in a humidified atmosphere of 95% air and 5% CO_2_ at 37°C. 293F cell lines were cultured in Free Style^TM^ Expression Medium without adding FBS. Peripheral blood mononuclear cells (PBMCs) derived from healthy human donors were provided by the Shanghai Blood Center. All cells were routinely tested for mycoplasma contamination.

### Expression and purification of GPC3/CD3 BiTE

GPC3/CD3 BiTE was constructed by standard DNA recombination technologies. The constructs were cloned into the expression vector pIH-hu9F2-CD3. 293F cells were transiently transfected with this expression vector and cultured in 293 freestyle medium and a humidified atmosphere of 95% air and 5% CO_2_ at 37°C in roller bottles, 135 rpm. After transfected cells were cultured for 6 days, cell supernatants containing the secreted proteins were collected and centrifuged. Then, the enriched supernatants were affinity-purified through a C-terminal 6×His tag and isolated by Ni Sepharose TM 6 Fast Flow. The column was equilibrated with buffer A (50 mM Na_2_HPO_4_, pH 7.0, 0.3 M NaCl) and the cell culture supernatant (300 mL) was applied to the column (10 mL) with a flow rate of 3 mL/min. The bound protein was eluted using a three-step gradient of buffer B (50 mM Na_2_HPO_4_, pH 7.0, 0.3 M NaCl, 0.5 M imidazole). The purity of GPC3/CD3 BiTE was estimated by SDS-PAGE and SEC-HPLC. All chemicals were of research grade and purchased from Sigma (Taufkirchen, Germany).

### Enzyme-linked immune-sorbent assay (ELISA)

After cell culture supernatants were collected and diluted with the culture medium, IFN-γ, TNF-e, IL-2, and IL-4 cytokines were analyzed by ELISA kit based on the protocol (Multi Sciences Biotechnology).

### Cytotoxicity of GPC3/CD3 BiTE effects on GPC3^+^ HCC cells *in vitro*

To determine whether GPC3/CD3 BiTE can recognize and kill GPC3-positive HCC cells, redirected cellular cytotoxicity was tested using human peripheral blood mononuclear cells (PBMCs) as effector cells. PBMCs were freshly isolated from healthy donors. Huh-7, HepG2, Hep3B, SK-Hep-1, and SK-Hep-1-GPC3 hepatocellular carcinoma cells were used as target cells. The target cells were incubated with PBMCs at effector cell:target cell ratios of 10:1, with varying concentrations of the GPC3/CD3 BiTE in 96-well plates for 18 h. Cytotoxicity analysis was performed using the CytoTox 96 ^®^Non-Radioactive Cytotoxicity Assay according to the manufacturer’s instructions. Each experiment was carried out using 5 replicated wells at the same GPC3/CD3 BiTE concentrations, and all the experiments were repeated 3 times.

### Western blot analysis

Cell lysates were harvested and centrifuged for 10 min at 13,000 rpm, 4°C. Proteins were separated on 10% SDS-PAGE gels and transferred to nitrocellulose membranes. Then, primary antibodies were incubated overnight at 4°C, and the secondary antibody was incubated for 2 h. Finally, the membrane was exposed by enhanced chemiluminescence reagents.

### The contribution of CD4^+^ and CD8^+^ T cells redirected to target cells by expressing granzyme B

Primary human CD4+ and CD8^+^ T cells were isolated from PBMCs by negative selection using a RosetteSep kit (Stem Cells Technology). Then, the CD4^+^/CD8^+^ T cells were stimulated by GPC3/CD3 BiTEs cocultured with HCC cells for 16 h. We detected the expression of granzyme B and the activation marker CD69 by FACS analysis.

### T cells redirected by GPC3/CD3 BiTE suppress of the tumorigenesis of subcutaneous GPC3^+^ Huh-7 xenografts

Six- to eight-week-old NOD/SCID (non-obese diabetic/severe combined immunodeficiency disease) mice were raised and treated under specific pathogen-free conditions. All animal experiments were carried out according to the protocols approved by the Shanghai Cancer Institute Experimental Animal Care Commission. Single-cell suspensions of HCC Huh-7 (2 × 10^6^) cells together with or without freshly isolated donor-derived PBMCs at a ratio of 1:2 (cancer cells:PBMCs) were injected subcutaneously on the right flank at a final volume of 0.2 mL/mouse. For the treatment model, six animals in each group were i.v. treated with PBS, 1 μg, or 10 μg of GPC3/CD3 BiTE, which started 1 h after cancer cell/PBMC mixture inoculation, and the treatment lasted for 10 consecutive days. Tumor dimensions were measured with calipers, and tumor volumes were calculated according to the formula V = 1/2 (length × width^2^).

To explore the antitumor activities of GPC3/CD3 BiTE in SK-Hep-1-GPC3 and SK-Hep-1 xenograft, the effector:target ratio was 1:2 (cancer cells:PBMCs). Mice in each of the three divided groups were treated with PBS, 1 μg, or 10 μg for 5 days. After observation for 36 days, the mice were sacrificed, and the tumors were obtained.

### Immunohistochemical (IHC) analysis

To assess the infiltration of T cells in the tumors, formalin-fixed paraffin-embedded tumor tissues were immunostained. The immunohistochemical staining procedures were performed as follows. After deparaffinization and rehydration, the tissue sections were incubated with 3% hydrogen peroxide in methanol to quench endogenous peroxidase. Then, the sections were heated in citrate buffer (pH 6.0) for 10 min in a water bath at 92∼98.5°C and were subsequently blocked for 30 min using bovine serum albumin (1%) at room temperature. The sections were incubated with an anti-CD3 antibody (Thermo Scientific RM-9107-S0) overnight at 4°C. Then, the sections were incubated with peroxidase-conjugated secondary antibodies (ChemMate™ DAKO EnVision™ Detection Kit, Peroxidase/DAB, Rabbit/Mouse, DAKO) for one hour and counterstained with hematoxylin.

### Statistical analysis

All data are presented as the means ± SD. For studies comparing two groups, the Student’s *t* test was used. For comparisons of more than two groups, we used one-way ANOVA with Bonferroni post-test. *P* < 0.05 (**) and *P* < 0.01 (***) were considered statistically significant.

## SUPPLEMENTARY FIGURES


