# Supplementary material for: Treatment of hepatocellular carcinoma with a GPC3-targeted bispecific T cell engager
Source: Oncotarget. 2017 May 16;8(32):52866–76. doi: 10.18632/oncotarget.17905 (PMC5581077; doi:10.18632/oncotarget.17905)
Supplement: Supplementary file 1 [file oncotarget-08-52866-s001.pdf]

Treatment of hepatocellular carcinoma with a GPC3-targeted bispecific T cell engager

SUPPLEMENTARY MATERIALS

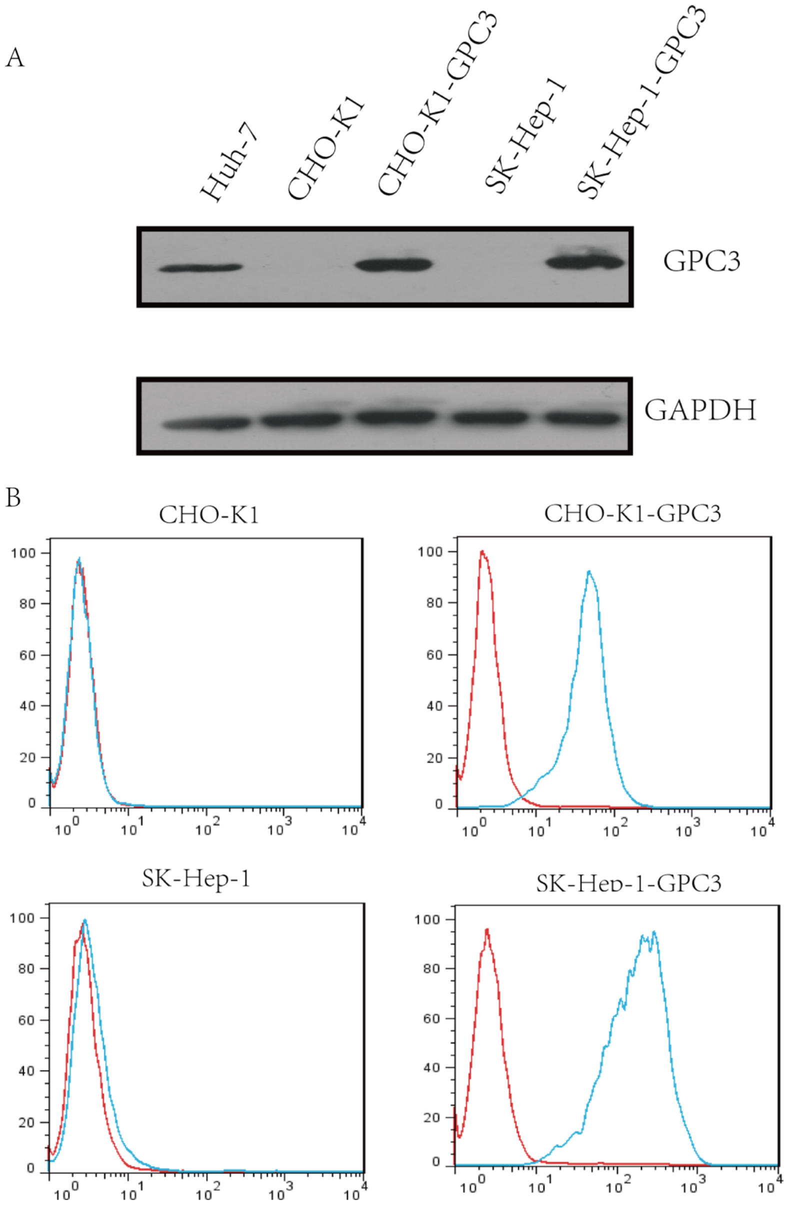

Supplementary Figure 1: Construction of the SK-Hep-1 GPC3 overexpression cell line. We tested the established overexpression cell lines by western blot (**A**) and FACS (**B**). The parent cells were the controls. CHO-K1 cells were the positive controls of the overexpression system.

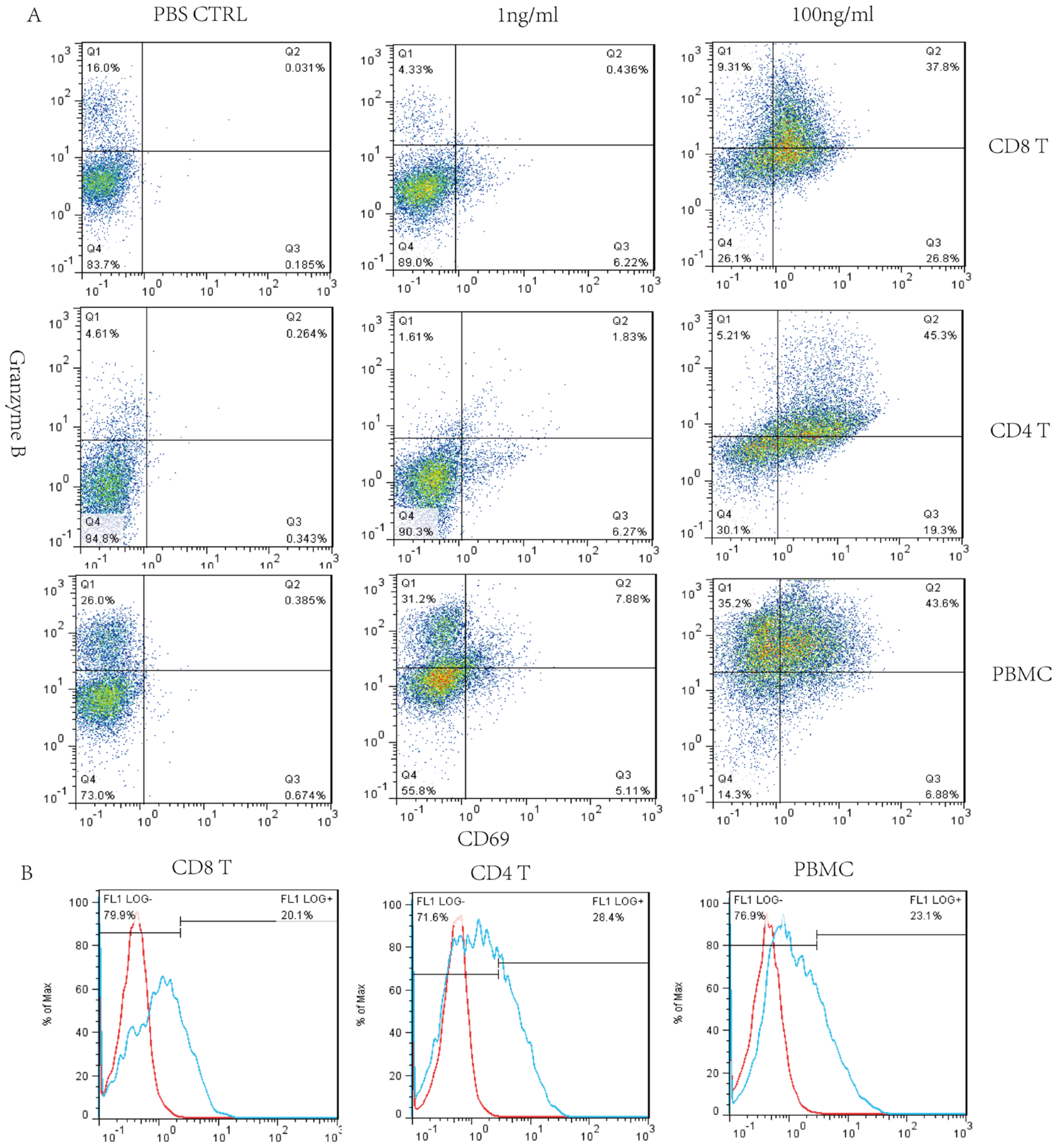

Supplementary Figure 2: Granzyme B expression when the GPC3/CD3 BiTE redirected lysis. Granzyme B expression when the GPC3/CD3 BiTE redirected lysis to HepG2 in the presence of $CD4^+$ and $CD8^+$ T cells. (**A**) Three-color flow cytometry analysis of granzyme B, PBMCs, $CD4^+$ and $CD8^+$ T cells separately cocultured with HepG2 cells in the presence of the GPC3/CD3 BiTE. (**B**) We detected the expression of the activation marker CD69 in permeabilized $CD4^+$ and $CD8^+$ T lymphocytes in response to 100 ng/mL of GPC3/CD3 BiTE for 16 h.

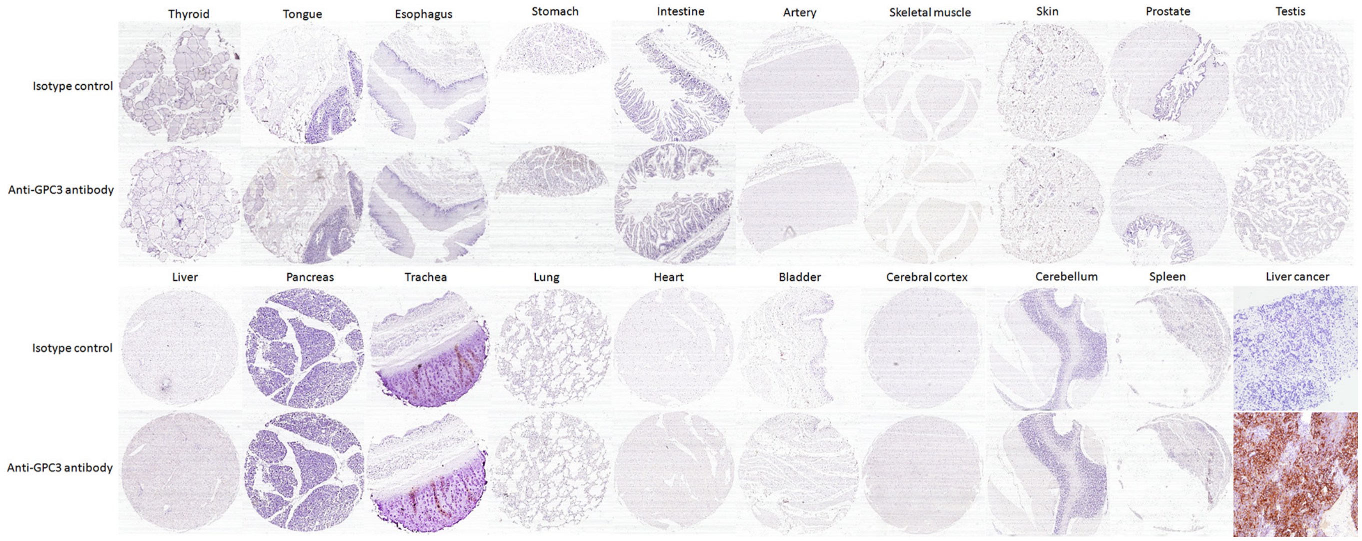

Supplementary Figure 3: GPC3 expression in normal human tissues. Tissue microarrays (TMAs) were immunostained with anti-GPC3 antibody (1G12). Sections of HCC with confirmed GPC3 expression were used as a positive control. The images were taken with a microscope (BX41, Olympus, PA) under ×200 magnification. An HOrg-N090-01 microarray (Outdo Biotech, Shanghai, China) containing 90 normal human tissue samples was immunostained using an anti-GPC3 antibody to determine the expression of GPC3. A rabbit IgG antibody served as an isotype control. The immunohistochemical staining procedures were performed as follows. After deparaffinization and rehydration, the sections were exposed to 3% H_2O_2 in methanol to eliminate endogenous peroxidase activity. Then, the sections were heated in citrate buffer (pH 6.0) for 10 min in a water bath at 92~98.5°C and were subsequently blocked for 30 min using bovine serum albumin (1%) at room temperature. The sections were incubated with a monoclonal anti-GPC3 antibody (mAb 1G12, BioMosaics Inc, Burlington, VT) overnight at 4°C. Then, the sections were incubated with peroxidase-conjugated secondary antibodies (ChemMate™ DAKO EnVision™ Detection Kit, Peroxidase/DAB, Rabbit/Mouse, DAKO) for one hour and counterstained with hematoxylin.
